# Supplementary material for: Level of Murine DDX3 RNA Helicase Determines Phenotype Changes of Hepatocytes In Vitro and In Vivo
Source: Int J Mol Sci. 2021 Jun 28;22(13):6958. doi: 10.3390/ijms22136958 (PMC8269429; doi:10.3390/ijms22136958)
Supplement: Supplementary file 1 [file ijms-22-06958-s001.zip › ijms-1188735-supplementary/suppl material/Supplementary Material.pdf]

Supplementary Table S1. List of DDX3 siRNA used in the study.

siRNA	Sequences, 5’→3’
#1	ccGuAuGcGuGAAAuuAuAdTsdT<br>uAuAAUUUcACGcAuACGGdTsdT
#2	uGcuGAAuGuAAAGAuuGAdTsdT<br>UcAAUCUUuAcAUUcAGcAdTsdT
#3	AcAcuuGucuuGAGAuAuudTsdT<br>AAuAUCUcAAGAcAAGUGUdTsdT
#4	AuGcGuGAAAuuAuAuAuudTsdT<br>AAuAuAuAAUUUcACGcAUdTsdT
#5	uGucuuGAGAuAuuAAcGAdTsdT<br>UCGUuAAuAUCUcAAGAcAdTsdT
#6	uGcAcAAGGuGucAAuucudTsdT<br>AGAAUUGAcACCUUGUGcAdTsdT
#7	GuGuGAGcuuuuAAuGuuAdTsdT<br>uAAcAUuAAAAGCUcAcACdTsdT
#8	AuuGGcGAcuuGAAAcuAAdTsdT<br>UuAGUUUcAAGUCGCcAAUdTsdT

control	cuuAcGcuGAGuAcuucGATsT<br>UCGAAGuACUcAGCGuAAGTsT

Upper case – ribonucleotide, lower case – 2′-O-methylribonucleotide, s –phosphorothioate group, T - thymidine.

Supplementary Table S2. List of primers used in the study.

Name	Forward, 5’→3’	Reverse, 5’→3’
GAPDH	TGCACCACCAACTGCTTAGC	GGCATGGACTGTGGTCATGAG
DDX3	CAACAGGCAACAACTGTCCTCCAC	GAATAGCATGCTTCTGCACTGGAGTTG
DBP	GGA AAC AGC AAG CCC AAA GAA	CAG CGG CGC AAA AAG ACT C
CCNE1	GTG GCT CCG ACC TTT CAG TC	CAC AGT CTT GTC AAT CTT GGC A
CDKN1	CCT GGT GAT GTC CGA CCT G	CCA TGA GCG CAT CGC AAT C
MCM3	AGC GCA GAG AGA CTA CTT GGA	CAG CCG ATA CTG ATT GTC ACT
CTNNB1	ATGGAGCCGGACAGAAAAGC	TGGGAGGTGTCAACATCTTCTT
GSK3	GCGTTCCCAAGAAGTGGCTTA	GGTCCAGCTTACGCATAATCTG
ELOVL3	TTC TCA CGC GGG TTA AAA ATG G	GAG CAA CAG ATA GAC GAC CAC
MMP10	GAG CCA CTA GCC ATC CTG G	CTG AGC AAG ATC CAT GCT TGG
C-MYC	GTC AAG AGG CGA ACA CAC AAC	TTG GAC GGA CAG GAT GTA TGC
TNF-A	AGC CCC CAG TCT GTA TCC TT	GGT CAC TGT CCC AGC ATC TT
PYGO-1	CCA TCG CGC TGA AGA GAG TTA	GTG TGT TGG CCT TGC GTT TT
PDK4	AGG GAG GTC GAG CTG TTC TC	GGA GTG TTC ACT AAG CGG TCA

Upper case – 2’-deoxynucleotide.

Supplementary Table S3. Characterization of LNP-siRNA used in the study.

	LNP size	siRNA concentration, ng/µl	siRNA entrapment efficiency

<table>
<tr><td>LNP-control</td><td>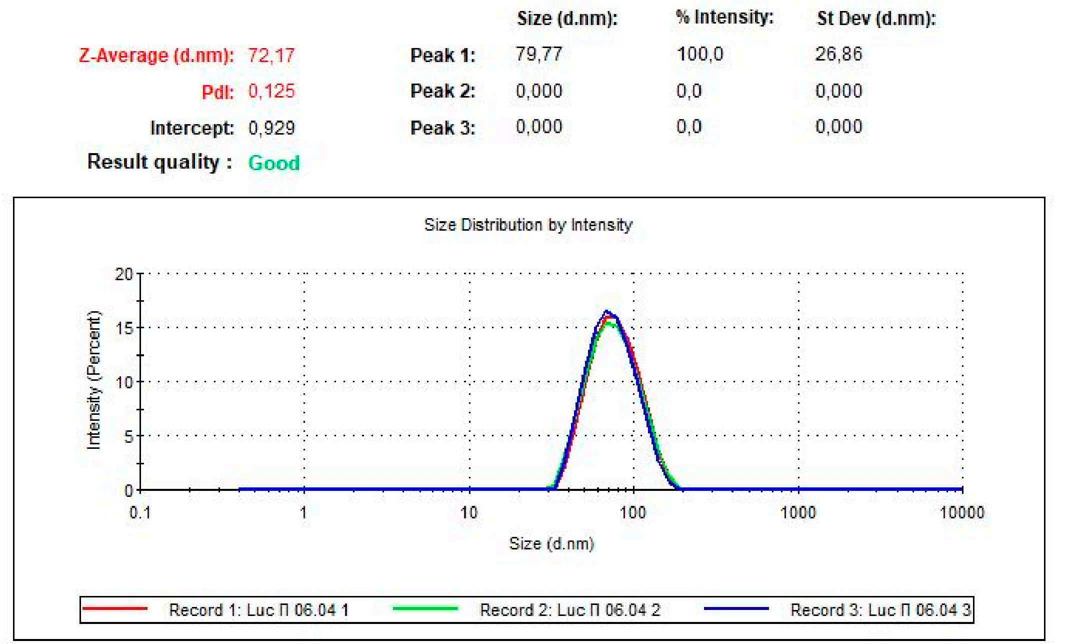
</td><td>137</td><td>63%</td></tr>
<tr><td>LNP-siRNA#5</td><td>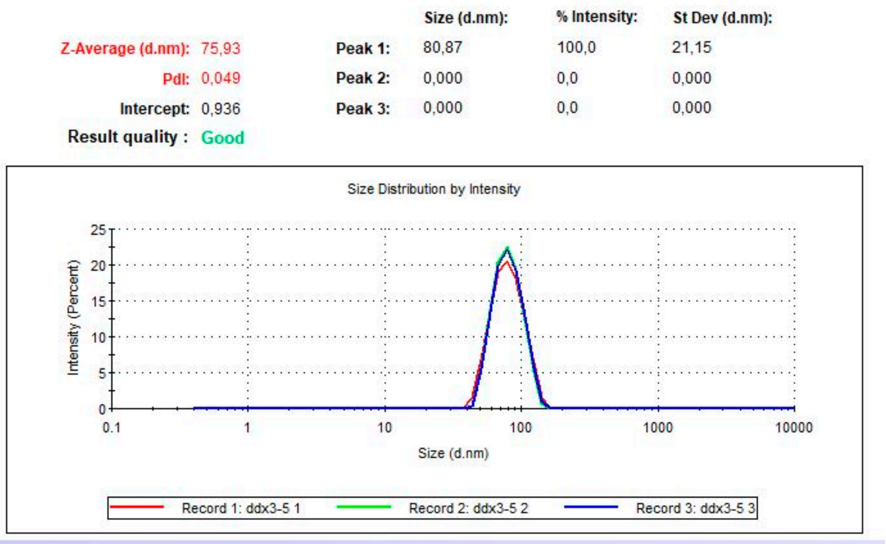
</td><td>123.5</td><td>57%</td></tr>
</table>

LNP-siRNA#7	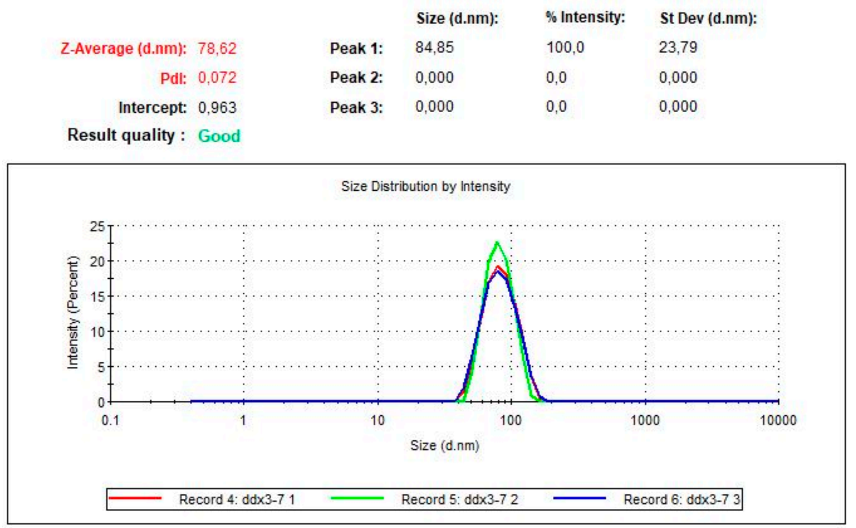	134.5	62%

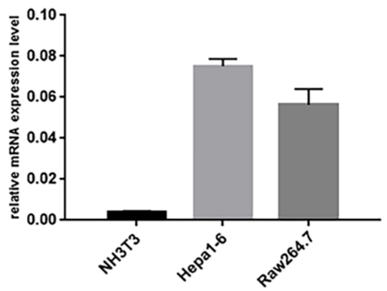

A.

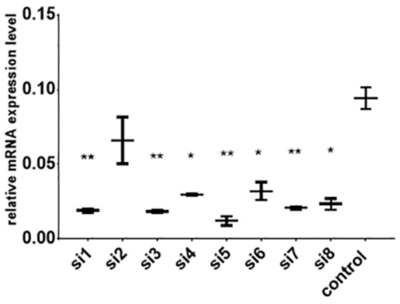

B.

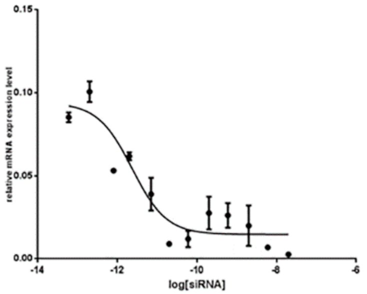

C.

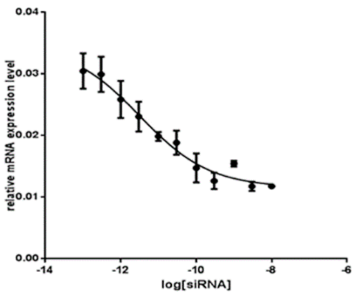

D.

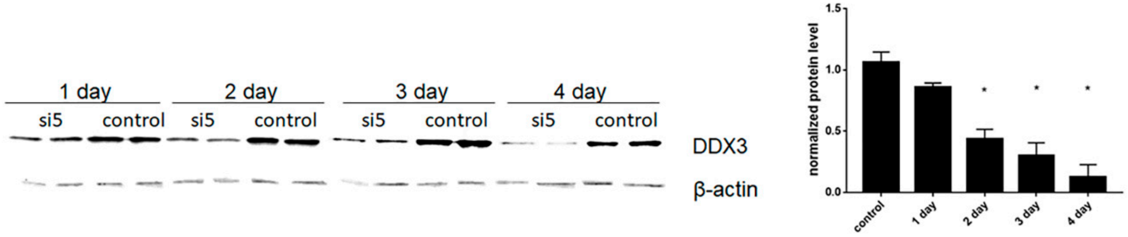

E.

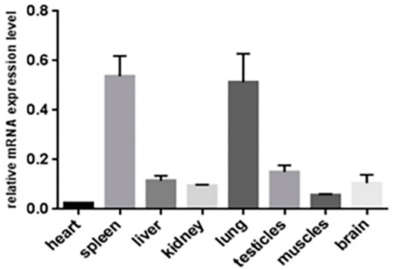

F.

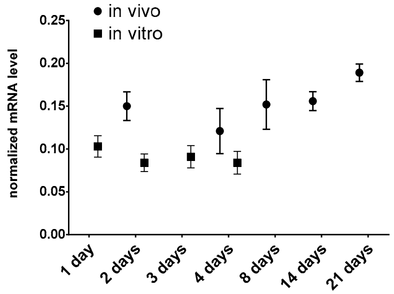

G.

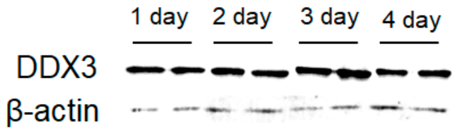

H.

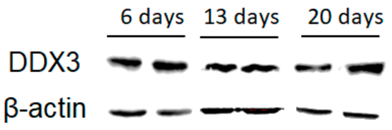

I.

Supplementary Figure S1. A. Determination of the DDX3 mRNA expression level in different cell lines by RT-qPCR. B. Determination of the DDX3 siRNA efficacy in Hepa1-6 cells by RT-qPCR (*$p<0.05$, **$p<0.01$). 1-8 are the numbers of siRNA from Suppl. table 1. Estimation IC50 for si#5 (C.) and si#7 (D.) in Hepa1-6 cells by RT-qPCR. E. Determination the efficacy of the DDX3 protein inhibition in Hepa1-6 cells by Western-blot. F. Estimation DDX3 mRNA expression level in different murine organs by RT-qPCR. G. Estimation DDX3 mRNA expression level at the different time points after

the siLuc treatment (control siRNA) *in vitro* and *in vivo* by RT-qPCR. Determination the DDX3 protein level at the different time points after siLuc (control siRNA) treatment *in vitro* (H.) and *in vivo* (I.) by Western-blot.

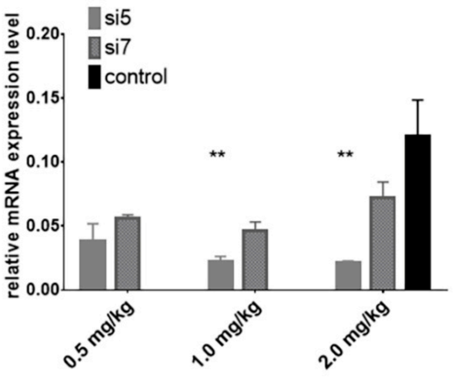

A.

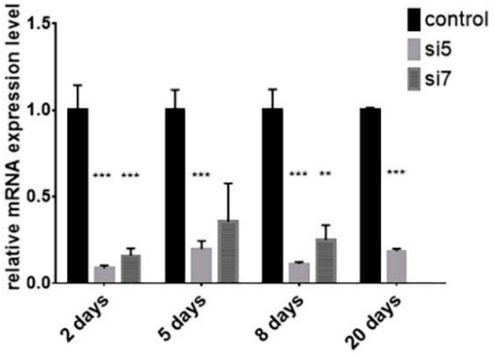

B.

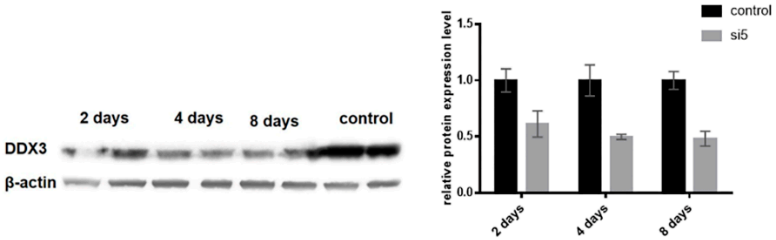

C.

Supplementary Figure S2. A. Estimation of the dose response for si#5 and si#7 by RT-qPCR in the murine liver. B. Determination of DDX3 mRNA recovery after RNAi-mediated inhibition by si#5 and si#7 by RT-qPCR. C. Determination the efficacy of DDX3 protein inhibition by Western-blot. *p<0.05, **p<0.01, ***p<0.001.

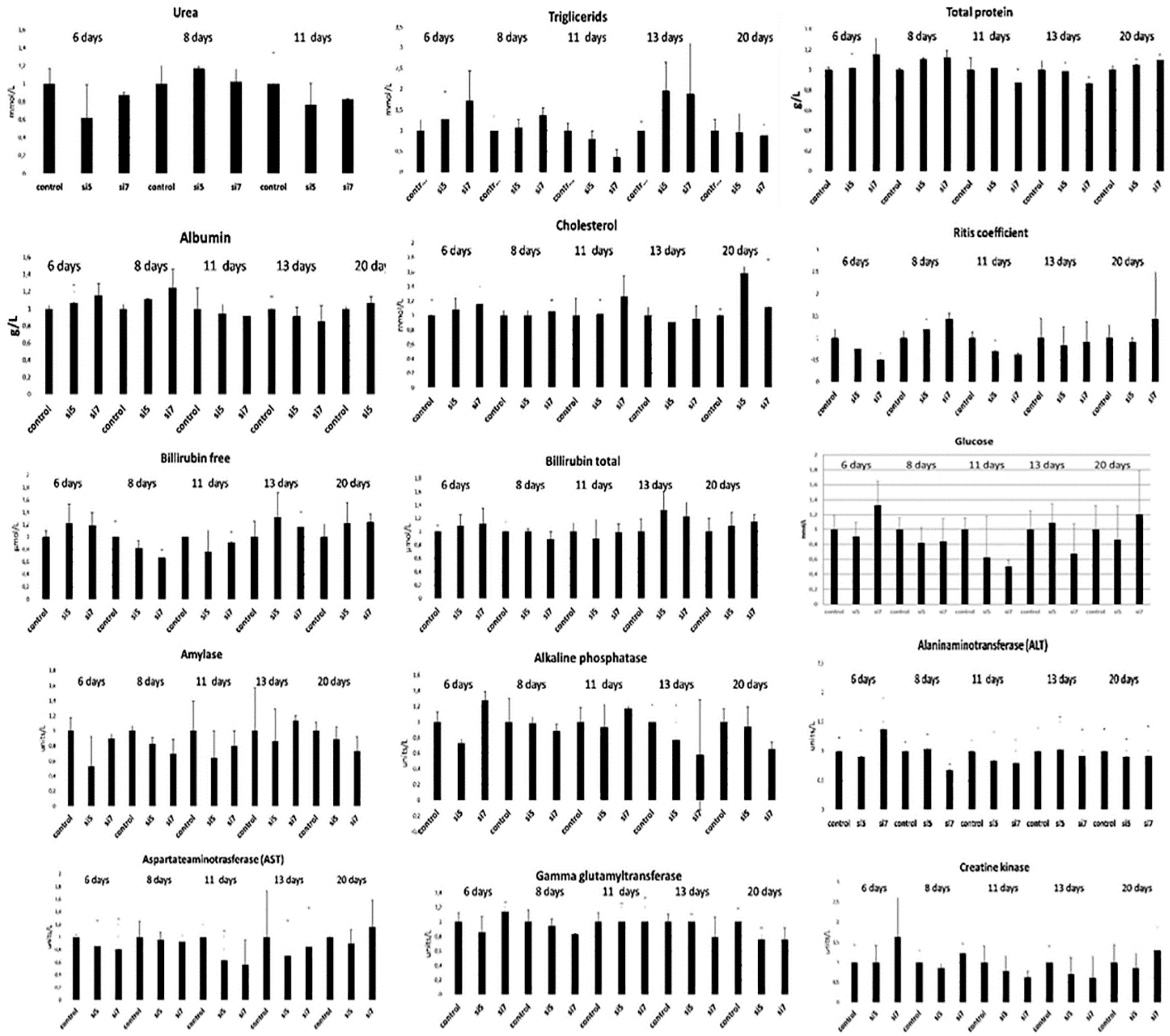

Supplementary Figure S3. Liver biochemical markers analyzed in animal blood after the administration of si#5 and si#7 biweekly for 3 weeks.

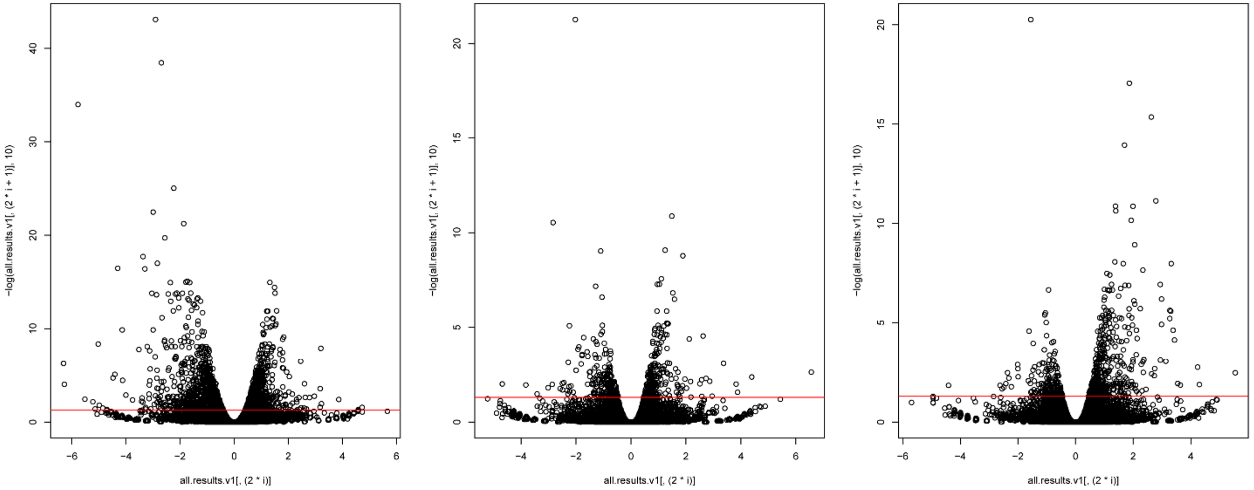

−log(all.results.v1[, (2 * i + 1)], 10)
all.results.v1[, (2 * i)]

A.

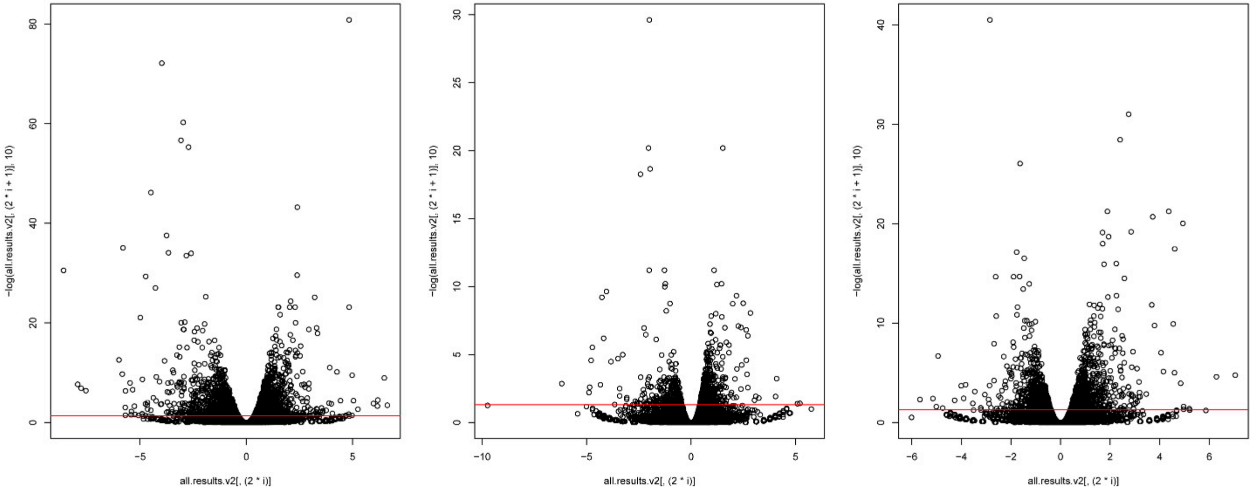

−log(all.results.v2[, (2 * i + 1)], 10)
all.results.v2[, (2 * i)]

B.

C.

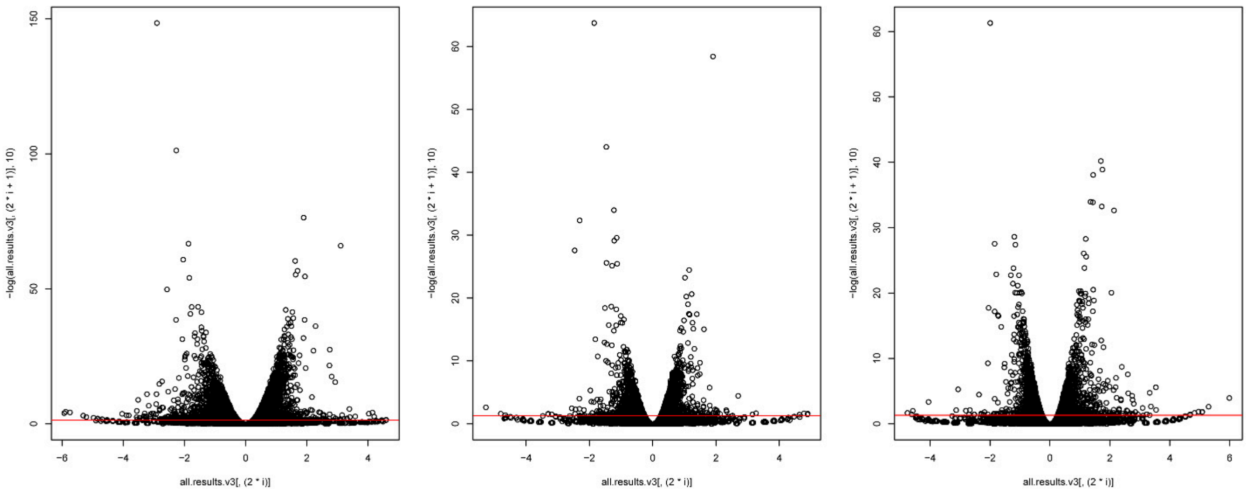

Supplementary Figure S4. Volcano plots of gene expression after DDX3 depletion by siRNA #5 and siRNA #7 in A. cells, B. liver day 6, C. liver day 13.

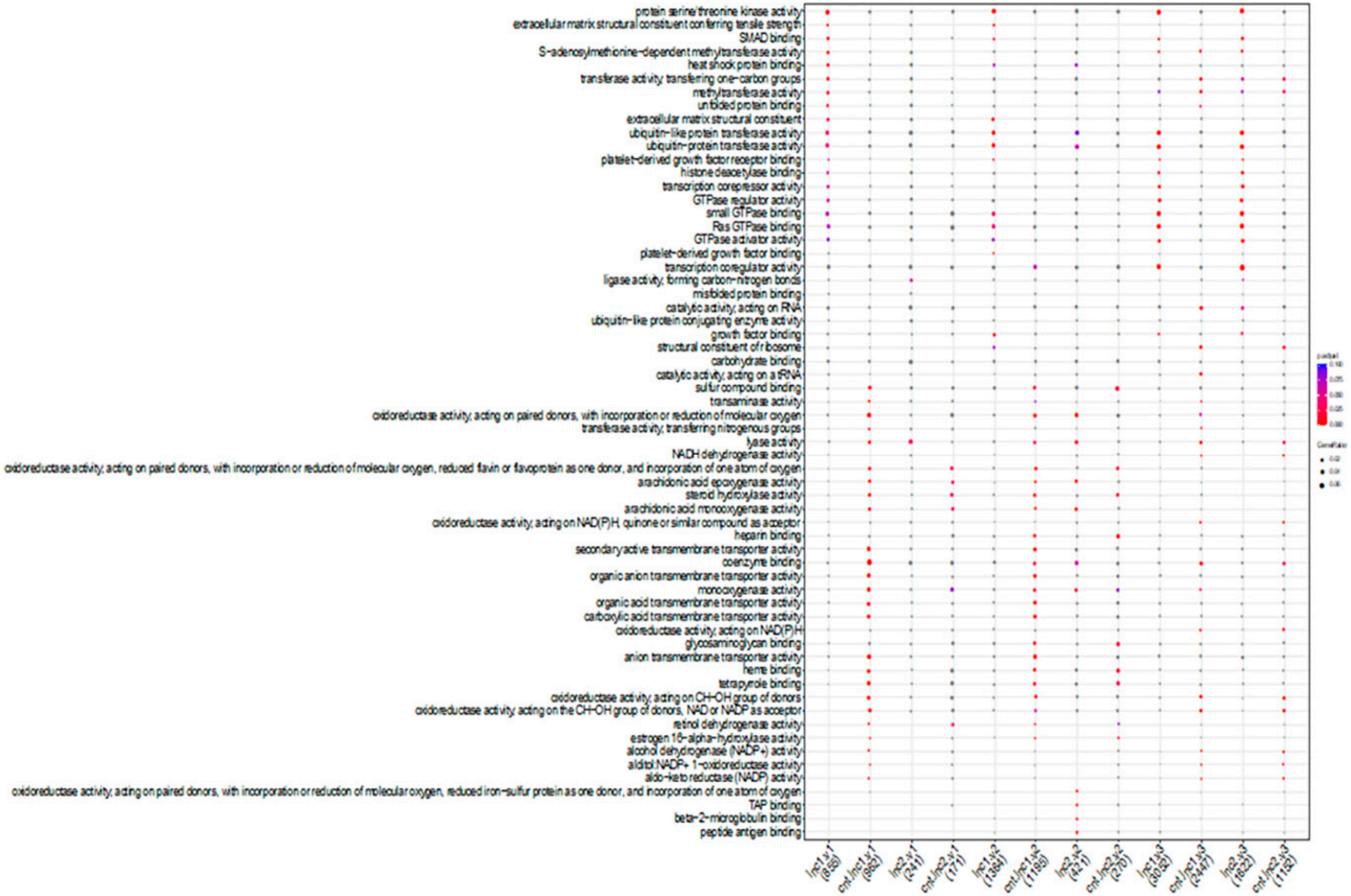
protein serine/threonine kinase activity
extracellular matrix structural constituent conferring tensile strength
SMAD binding
S-adenosylmethionine-dependent methyltransferase activity
heat shock protein binding
transferase activity, transferring one-carbon groups
methyltransferase activity
unfolded protein binding
extracellular matrix structural constituent
ubiquitin-like protein transferase activity
ubiquitin-protein transferase activity
platelet-derived growth factor receptor binding
histone deacetylase binding
transcription corepressor activity
GTPase regulator activity
small GTPase binding
Ras GTPase binding
GTPase activator activity
platelet-derived growth factor binding
transcription coregulator activity
ligase activity, forming carbon-nitrogen bonds
misfolded protein binding
catalytic activity, acting on RNA
ubiquitin-like protein conjugating enzyme activity
growth factor binding
structural constituent of ribosome
carbohydrate binding
catalytic activity, acting on a tRNA
sulfur compound binding
transaminase activity
oxidoreductase activity, acting on paired donors, with incorporation or reduction of molecular oxygen
transferase activity, transferring nitrogenous groups
lyase activity
NADH dehydrogenase activity
oxidoreductase activity, acting on paired donors, with incorporation or reduction of molecular oxygen, reduced flavin or flavoprotein as one donor, and incorporation of one atom of oxygen
arachidonic acid epoxygenase activity
steroid hydroxylase activity
arachidonic acid monooxygenase activity
oxidoreductase activity, acting on NAD(P)H, quinone or similar compound as acceptor
heparin binding
secondary active transmembrane transporter activity
coenzyme binding
organic anion transmembrane transporter activity
monooxygenase activity
organic acid transmembrane transporter activity
carboxylic acid transmembrane transporter activity
oxidoreductase activity, acting on NAD(P)H
glycosaminoglycan binding
anion transmembrane transporter activity
heme binding
tetrapyrrole binding
oxidoreductase activity, acting on CH-OH group of donors
oxidoreductase activity, acting on the CH-OH group of donors, NAD or NADP as acceptor
retinol dehydrogenase activity
estrogen 16-alpha-hydroxylase activity
alcohol dehydrogenase (NADP+) activity
alditol:NADP+ 1-oxidoreductase activity
aldo-keto reductase (NADP) activity
oxidoreductase activity, acting on paired donors, with incorporation or reduction of molecular oxygen, reduced iron-sulfur protein as one donor, and incorporation of one atom of oxygen
TAP binding
beta-2-microglobulin binding
peptide antigen binding
lnc1.y1 (858)
cnt.lnc1.y1 (982)
lnc2.y1 (241)
cnt.lnc2.y1 (171)
lnc1.y2 (1364)
cnt.lnc1.y2 (1195)
lnc2.y2 (421)
cnt.lnc2.y2 (270)
lnc1.y3 (3052)
cnt.lnc1.y3 (2447)
lnc2.y3 (1622)
cnt.lnc2.y3 (1152)
p.adjust
0.100
0.075
0.050
0.025
0.000
GeneRatio

A.

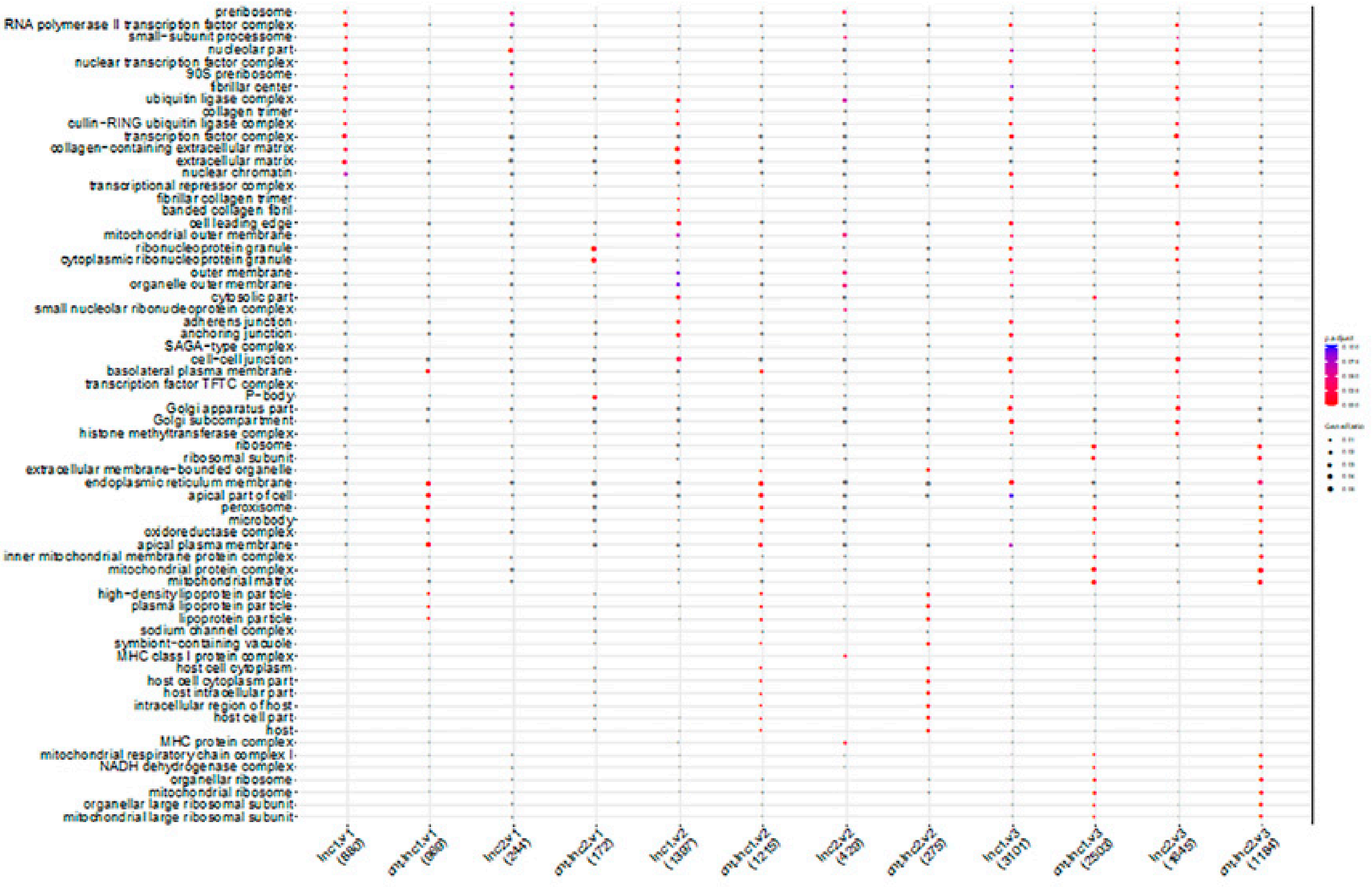

preribosome
RNA polymerase II transcription factor complex
small-subunit processome
nucleolar part
nuclear transcription factor complex
90S preribosome
fibrillar center
ubiquitin ligase complex
collagen trimer
cullin-RING ubiquitin ligase complex
transcription factor complex
collagen-containing extracellular matrix
extracellular matrix
nuclear chromatin
transcriptional repressor complex
fibrillar collagen trimer
banded collagen fibril
cell leading edge
mitochondrial outer membrane
ribonucleoprotein granule
cytoplasmic ribonucleoprotein granule
outer membrane
organelle outer membrane
cytosolic part
small nucleolar ribonucleoprotein complex
adherens junction
anchoring junction
SAGA-type complex
cell-cell junction
basolateral plasma membrane
transcription factor TFTC complex
P-body
Golgi apparatus part
Golgi subcompartment
histone methyltransferase complex
ribosome
ribosomal subunit
extracellular membrane-bounded organelle
endoplasmic reticulum membrane
apical part of cell
peroxisome
microbody
oxidoreductase complex
apical plasma membrane
inner mitochondrial membrane protein complex
mitochondrial protein complex
mitochondrial matrix
high-density lipoprotein particle
plasma lipoprotein particle
lipoprotein particle
sodium channel complex
symbiont-containing vacuole
MHC class I protein complex
host cell cytoplasm
host cell cytoplasm part
host intracellular part
intracellular region of host
host cell part
host
MHC protein complex
mitochondrial respiratory chain complex I
NADH dehydrogenase complex
organellar ribosome
mitochondrial ribosome
organellar large ribosomal subunit
mitochondrial large ribosomal subunit
lnc1.v1
cnj.lnc1.v1
lnc2.v1 (244)
cnj.lnc2.v1 (172)
lnc1.v2 (1397)
cnj.lnc1.v2 (1215)
lnc2.v2 (420)
cnj.lnc2.v2 (275)
lnc1.v3 (3101)
cnj.lnc1.v3 (2503)
lnc2.v3
cnj.lnc2.v3 (1184)

B.

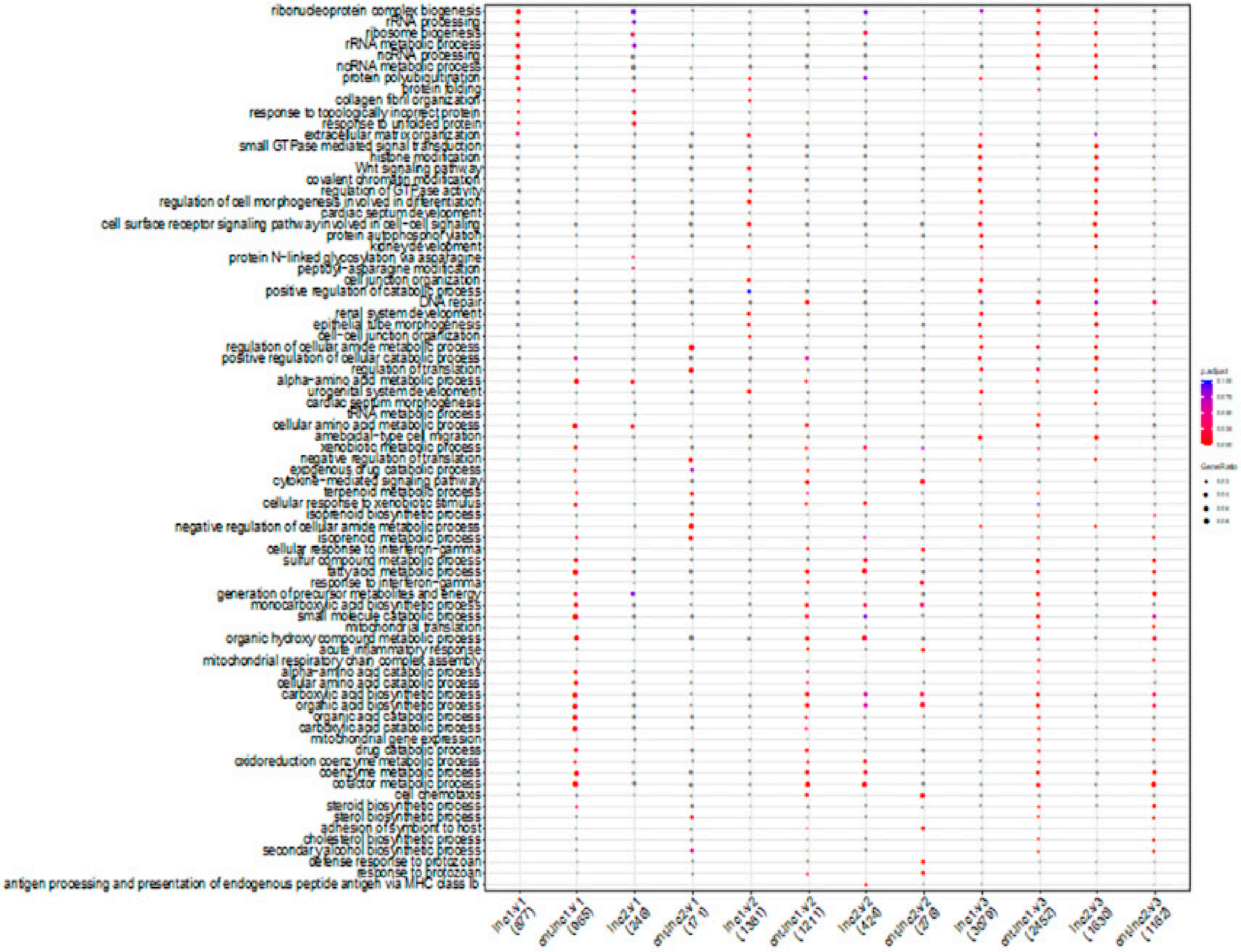

ribonucleoprotein complex biogenesis
rRNA processing
ribosome biogenesis
rRNA metabolic process
ncRNA processing
ncRNA metabolic process
protein polyubiquitination
protein folding
collagen fibril organization
response to topologically incorrect protein
response to unfolded protein
extracellular matrix organization
small GTPase mediated signal transduction
histone modification
Wnt signaling pathway
covalent chromatin modification
regulation of GTPase activity
regulation of cell morphogenesis involved in differentiation
cardiac septum development
cell surface receptor signaling pathway involved in cell−cell signaling
protein autophosphorylation
kidney development
protein N−linked glycosylation via asparagine
peptidyl−asparagine modification
cell junction organization
positive regulation of catabolic process
DNA repair
renal system development
epithelial tube morphogenesis
cell−cell junction organization
regulation of cellular amide metabolic process
positive regulation of cellular catabolic process
regulation of translation
alpha−amino acid metabolic process
urogenital system development
cardiac septum morphogenesis
tRNA metabolic process
cellular amino acid metabolic process
ameboidal−type cell migration
xenobiotic metabolic process
negative regulation of translation
exogenous drug catabolic process
cytokine−mediated signaling pathway
terpenoid metabolic process
cellular response to xenobiotic stimulus
isoprenoid biosynthetic process
negative regulation of cellular amide metabolic process
isoprenoid metabolic process
cellular response to interferon−gamma
sulfur compound metabolic process
fatty acid metabolic process
response to interferon−gamma
generation of precursor metabolites and energy
monocarboxylic acid biosynthetic process
small molecule catabolic process
mitochondrial translation
organic hydroxy compound metabolic process
acute inflammatory response
mitochondrial respiratory chain complex assembly
alpha−amino acid catabolic process
cellular amino acid catabolic process
carboxylic acid biosynthetic process
organic acid biosynthetic process
organic acid catabolic process
carboxylic acid catabolic process
mitochondrial gene expression
drug catabolic process
oxidoreduction coenzyme metabolic process
coenzyme metabolic process
cofactor metabolic process
cell chemotaxis
steroid biosynthetic process
sterol biosynthetic process
adhesion of symbiont to host
cholesterol biosynthetic process
secondary alcohol biosynthetic process
defense response to protozoan
response to protozoan
antigen processing and presentation of endogenous peptide antigen via MHC class Ib
p.adjust
GeneRatio

C.

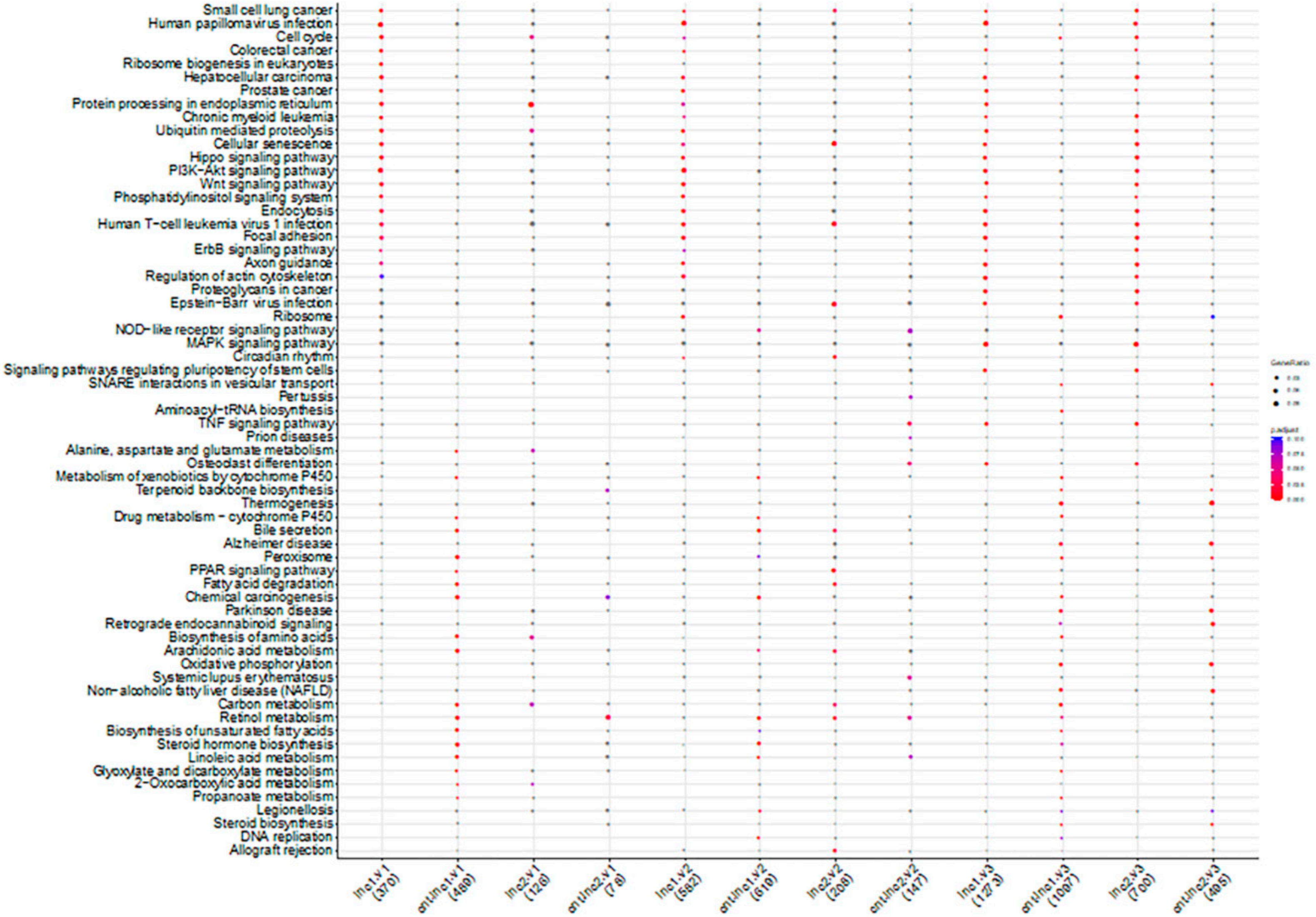

Small cell lung cancer
Human papillomavirus infection
Cell cycle
Colorectal cancer
Ribosome biogenesis in eukaryotes
Hepatocellular carcinoma
Prostate cancer
Protein processing in endoplasmic reticulum
Chronic myeloid leukemia
Ubiquitin mediated proteolysis
Cellular senescence
Hippo signaling pathway
PI3K−Akt signaling pathway
Wnt signaling pathway
Phosphatidylinositol signaling system
Endocytosis
Human T−cell leukemia virus 1 infection
Focal adhesion
ErbB signaling pathway
Axon guidance
Regulation of actin cytoskeleton
Proteoglycans in cancer
Epstein−Barr virus infection
Ribosome
NOD−like receptor signaling pathway
MAPK signaling pathway
Circadian rhythm
Signaling pathways regulating pluripotency of stem cells
SNARE interactions in vesicular transport
Pertussis
Aminoacyl−tRNA biosynthesis
TNF signaling pathway
Prion diseases
Alanine, aspartate and glutamate metabolism
Osteoclast differentiation
Metabolism of xenobiotics by cytochrome P450
Terpenoid backbone biosynthesis
Thermogenesis
Drug metabolism − cytochrome P450
Bile secretion
Alzheimer disease
Peroxisome
PPAR signaling pathway
Fatty acid degradation
Chemical carcinogenesis
Parkinson disease
Retrograde endocannabinoid signaling
Biosynthesis of amino acids
Arachidonic acid metabolism
Oxidative phosphorylation
Systemic lupus erythematosus
Non−alcoholic fatty liver disease (NAFLD)
Carbon metabolism
Retinol metabolism
Biosynthesis of unsaturated fatty acids
Steroid hormone biosynthesis
Linoleic acid metabolism
Glyoxylate and dicarboxylate metabolism
2−Oxocarboxylic acid metabolism
Propanoate metabolism
Legionellosis
Steroid biosynthesis
DNA replication
Allograft rejection
lnc1.v1 (370)
antilnc1.v1 (469)
lnc2.v1 (126)
antilnc2.v1 (78)
lnc1.v2 (582)
antilnc1.v2 (610)
lnc2.v2 (208)
antilnc2.v2 (147)
lnc1.v3 (1273)
antilnc1.v3 (1007)
lnc2.v3 (700)
antilnc2.v3 (495)
GeneRatio
p.adjust

D.

Supplementary Figure S5. Gene ontology analysis of gene expression after DDX3 depletion (KD) by siRNA #5 and siRNA #7: A. molecular functions (MF), B. Cellular components (CC), C. Biological pathways (BP), D. KEGG. v1 marked liver samples at 6th day of DDX3 KD, v2 – liver samples at 13th day of DDX3 KD, v3 – cells after DDX3 KD. Lnc1 is siRNA #5, lnc2 – siRNA #7, cntrl – control cells.
